# An Instrumented Cochlea Model for the Evaluation of Cochlear Implant Electrical Stimulus Spread

**DOI:** 10.1109/TBME.2021.3059302

**Published:** 2021-02-15

**Authors:** Chen Jiang, Shreya Singhal, Thomas Landry, Iwan V. Roberts, Simone R. de Rijk, Tim Brochier, Tobias Goehring, Yu C. Tam, Robert P. Carlyon, George G. Malliaras, Manohar L. Bance

**Affiliations:** 1 Cambridge Hearing Group, Department of Clinical Neurosciences, Clifford Allbutt Building, Cambridge Biomedical CampusUniversity of Cambridge 2152 CB2 0AH Cambridge U.K.; 2 Department of Electronic EngineeringTsinghua University 12442 Beijing 100084 China; 3 Electrical Engineering Division, Department of EngineeringUniversity of Cambridge 2152 CB3 0FA Cambridge U.K.; 4 Cambridge Hearing Group, Department of Clinical Neurosciences, Clifford Allbutt Building, Cambridge Biomedical CampusUniversity of Cambridge 2152 CB2 0AH Cambridge U.K.

**Keywords:** Cochlear implants, electrical stimulus spread, cochlea model, 3D printing

## Abstract

Cochlear implants use electrical stimulation of the auditory nerve to restore the sensation of hearing to deaf people. Unfortunately, the stimulation current spreads extensively within the cochlea, resulting in “blurring” of the signal, and hearing that is far from normal. Current spread can be indirectly measured using the implant electrodes for both stimulating and sensing, but this provides incomplete information near the stimulating electrode due to electrode-electrolyte interface effects. Here, we present a 3D-printed “unwrapped” physical cochlea model with integrated sensing wires. We integrate resistors into the walls of the model to simulate current spread through the cochlear bony wall, and “tune” these resistances by calibration with an *in-vivo* electrical measurement from a cochlear implant patient. We then use this model to compare electrical current spread under different stimulation modes including monopolar, bipolar and tripolar configurations. Importantly, a trade-off is observed between stimulation amplitude and current focusing among different stimulation modes. By combining different stimulation modes and changing intracochlear current sinking configurations in the model, we explore this trade-off between stimulation amplitude and focusing further. These results will inform clinical strategies for use in delivering speech signals to cochlear implant patients.

## I. Introduction

Cochlear implants (CIs) are considered life-changing devices for the rehabilitation of severe-to-profound hearing loss [1], [2]. However, the restored hearing function is far from normal. Most CI users’ speech comprehension breaks down in challenging listening conditions with background noise, and music is poorly appreciated [3], [4]. Additionally, a small, but significant proportion of patients perform poorly for speech comprehension even in quiet environments [5]. Despite CIs having up to 26 intracochlear electrodes that can be used for the stimulation [6], traditionally only between 4-8 independent channels of information have been reported [7], [8]. These issues can be largely attributed to current spread, that results in “blurring” of the input signal at the neuronal level [9]. The importance of current is seen not just in cochlear implants but also in other neural prostheses that require independent spatial channels for optimal performance, rather than just time domain parameters such as stimulation rate [10], [11].

To manipulate current spread, different stimulation modes have been used, i.e., the relative spatial locations of the current source and the current sink [12]. Typical stimulation modes include monopolar (MP), bipolar (BP), and tripolar (TP) modes [2], [13]. In addition, mixtures of these modes can also be used, such as TP with some percentage of the current sunk intracochlearly, and the rest returning to an extracochlear ground on the casing (called partial tripolar (pTP)), and similarly with BP stimulation [14]–[16]. Similar current steering methods have been also explored in neural prostheses, for example current steering in spinal cord stimulation [17].

To understand how stimulation current spreads inside the cochlea, researchers have measured the current spread-induced voltage (SIV) signals *in-vivo* in both humans and animal models using CIs as recording devices [18], [19]. Injecting current on one electrode causes current spread inside the cochlear fluids, and results in a voltage being expressed on other electrodes, which is a function of several parameters, such as the distance from the stimulating electrode, and the impedance to current flow out of the cochlea, both through the walls (transverse impedance) and along the cochlear fluids (longitudinal impedance) [20]. This SIV relative to the ground electrode can be measured and reported in living patients, as CIs are capable of “back telemetry”, i.e., reporting measured intracochlear parameters back to interrogating software. These measurements are available in clinical software of some cochlear implant companies, for instance as the trans-impedance matrix (TIM) for Cochlear Corp devices, impedance field telemetry (IFT) for MEDEL Corp, or the electrical field imaging (EFI) matrix for Advanced Bionics devices [21]. In these measurements, CIs are used as both stimulators and recorders. However, these measurements cannot reveal the whole distribution of the SIV in the cochlea. This is because the recorded voltage signal measured from a stimulating electrode contains a considerable voltage component induced at the electrode-tissue/fluid interface, which is unstable over time, and does not reveal the true voltage in the fluid a few micrometers away from the interface. In other words, only the measurements from the non-stimulating electrodes are reliable, as they are measured with essentially no current flow, using high-impedance amplifiers. Hence, there is missing data at the location of the stimulating electrode, which is in fact the most important measurement point to characterize how spatially focused the stimulus is at each electrode. In addition to the *in-vivo* investigations, there have been *in-vitro* studies [19], [22] using these types of measurements, but for which the same problem remains. It is essential to separate the stimulating electrodes and the sensing/recording electrodes to obtain the full distribution of the SIV. Although Computational models have been used to predict current spread in the cochlea, several assumptions and simplifications are made to make the solutions tractable (see more discussion about the comparison between computational and physical models in Supplementary Materials). Complementary to previous modelling work on CIs [23]–[25], we propose a novel *in-vitro* model approach in this paper, by separating the stimulating and sensing/recording electrodes to obtain the full distribution of the SIV.

In this study, we developed a 3D-printed “unwrapped” artificial cochlea with 14 instrumented sensing electrodes to measure the SIV signals along the cochlea. By “unwrapped”, we mean the snail shape of the cochlea has been reduced to a linear structure, whilst keeping the dimensions and their gradual changes from basal to apical turns similar to those in the human cochlea. Importantly, the voltage measurement locations were placed on the cochlear wall of the stimulated cochlea, and hence occupy a similar location to where the spiral ganglion cells would be in the human cochlea with roughly the same distance between stimulating electrodes and receiving receptors as would occur from electrode to spiral ganglion cells. Furthermore, the recording electrodes do not take up any intracochlear volume, so that the volume of the artificial “perilymph” (in this case saline) is not changed or its electrical characteristics altered. We measured the SIV distribution along the cochlea and compared the SIV distributions under different CI stimulation modes, including MP, BP, TP, and pTP, as well as BP+*n* and TP+*n* modes. The key research questions were: how to optimize a 3D-printed *in-vitro* cochlea model so that it mimics a living cochlea; how the SIV is distributed under different stimulation modes using intra- and/or extracochlear current sinking electrodes; how the different configurations in these stimulation modes affect current spread; and, whether we can find compromises for some trade-offs to potentially optimize CI performance. The ultimate benefit of optimizing a 3D-printed in-vitro cochlea model is the potential ability to rapidly perform studies on multiple types of stimulation strategies and their effects on the electric fields inside the cochlea, without the long processing time required for computational models, and with the complex electrode-electrolyte interface built into the model, which can be difficult to computationally account for.

## II. Methods

### A. 3D-Printed Unwrapped Cochlea

**Fig. 1.**
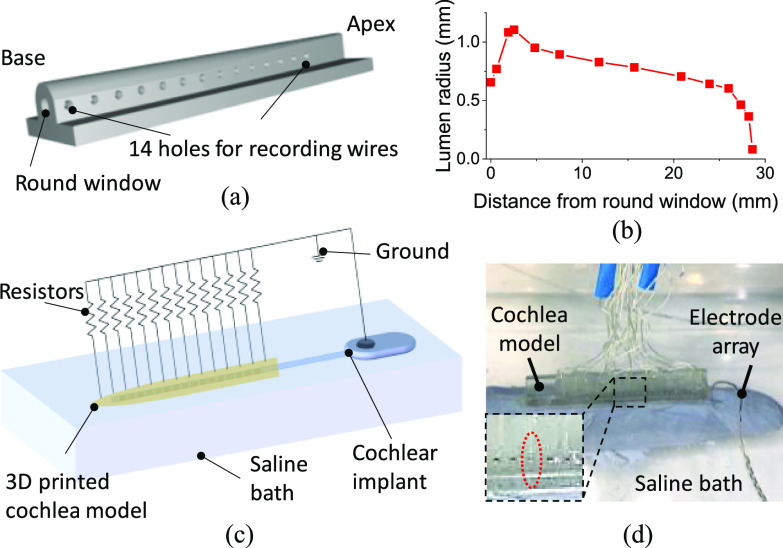
(a) The 3D schematic model of the 3D-printed unwrapped cochlea. (b) The tapering geometry of the cochlea lumen, with the size of lumen as a function of the distance from the round window. (c) A schematic of the experimental setup for the spread-induced voltage (SIV) measurements. (d) A photo of the CI “implanted” into the 3D printed cochlea model. Inset shows alignment of the 8^th^ electrode (CI) and the 8^th^ wire (cochlea model).

The 3D model of an unwrapped cochlea (cochlear duct was uncurled to form a linear structure, rather than a complex 3D spiral structure) was designed using Solidworks 2018. The lumen geometry had a circular cross-section with varying diameter along its length according to a previously published measurement of the cross-sectional area in a human cochlea [26] (Fig. 1(b)). The lumen is tapered with a larger diameter at the base and smaller diameter at the apex (Fig. 1(b)). The model was 3D printed with clear electrically-insulating methacrylate resin and ultraviolet (UV)-cured using a Formlabs Form 2 3D printer. Note that three scalas were combined together to form the lumen diameter. Teflon coated silver wires (World Precision Instruments AGT1010) were inserted through the model wall every 2 mm, starting 1 mm from the basal opening, and affixed with manually applied UV-cured adhesive (Dymax Multi-Cure 9-911-REV-B). The 2-mm wire spacing was designed to balance the trade-off between the difficulty of close manual wire insertion and density required to reveal SIV distributions. Although the spacing is larger than the electrode spacing of the CI used and so less densely sampling, the wires would record the voltage distributions under different stimulation modes more accurately and precisely, and would actually be more dense sampling than using implant electrodes for pTP+*n* modes, under which the SIV distributions recorded by a CI would be imprecise over 3 mm using Advanced Bionics devices (of which the electrode pitch is about 1 mm), as several adjacent electrodes are stimulating and cannot record accurately. Wire depths were individually gauged based on a micro-computed tomography scan, and intended to be just at lumen surface level, which was used as an approximation to the voltage measurements at the Rosenthal's canal (where the auditory nerve cell bodies are located in human cochleae). Teflon coating was used to avoid cross-talk between any two wires when being immersed in saline. The end of the wires facing the cochlea lumen were chlorinated to reduce interface impedance. The apex of the cochlea had a polyethylene tubing with inner diameter of 0.011 inch and outer diameter of 0.024 inch (BD Intramedic PE10), attached with the same UV-cure adhesive as for the wires, to allow saline solution flushing through the cochlea from the apex, while ensuring that no air bubbles were trapped (Fig. S2). In our model, we simplified and combined all the current pathways into the resistors (aside from the inevitable apical and basal fluid channels) in our artifical cochlea model. Note that we did not include Rosenthal's canal and a basilar membrane in our model, for which the reasons can be found in Supplementary Materials.

### B. CI, Stimuli and EFI

The HiFocus 1J CI electrode by Advanced Bionics [27] was used in this study. It is a platinum-iridium alloy 16-electrode intracochlear array, housed within a silicone carrier, with an electrode lead fantail extending to the titanium case electronics. The electrodes are embedded on the medial surface of the implant and are numbered 1 to 16 from apex to base. Mapping was done by aligning electrode 8 (CI) and recording wire 8 (artificial cochlea), as shown in Fig. 1(d), and scaling the remaining data points according to the relative geometry between the CI and the artificial cochlea wires.

**Fig. 2.**
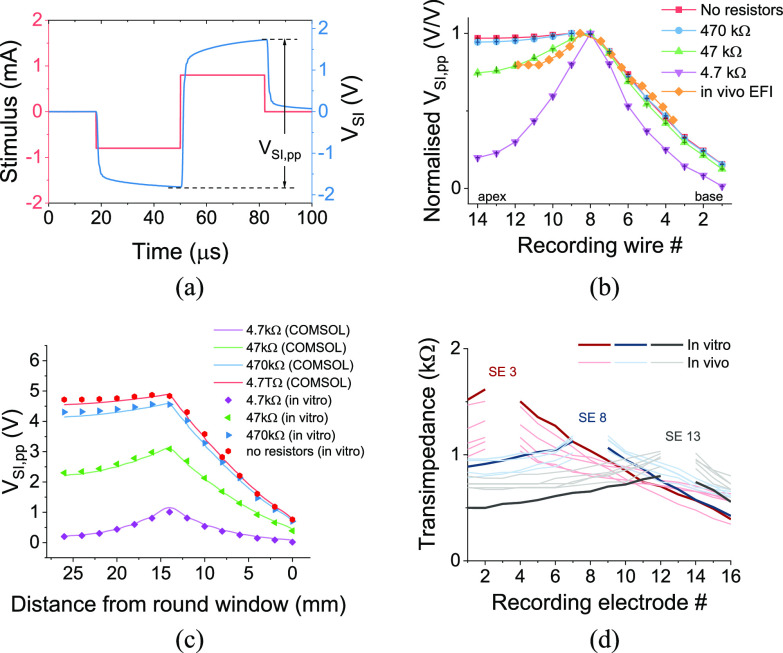
(a) The current stimulus injected into the cochlea as a function of time and the measured SIV, indicating the peak-to-peak SIV (*V*_SI,pp_) measured at a recording electrode situated in the model wall. (b) The effect of cross-wall resistors on SIV distribution in the artificial cochlea. The measured *V*_SI,pp_ was normalised and compared with an *in-vivo* patient EFI profile. (c) The validation of cross-wall resistors by comparing the artificial *in-vitro* model with a computational COMSOL model. (d) The comparison of EFI data between the *in-vitro* model (the average of three measurements) and *in-vivo* patients (collected intra-operatively) under stimulations from electrodes 3, 8 and 13; SE: stimulating electrode.

**Fig. 3.**
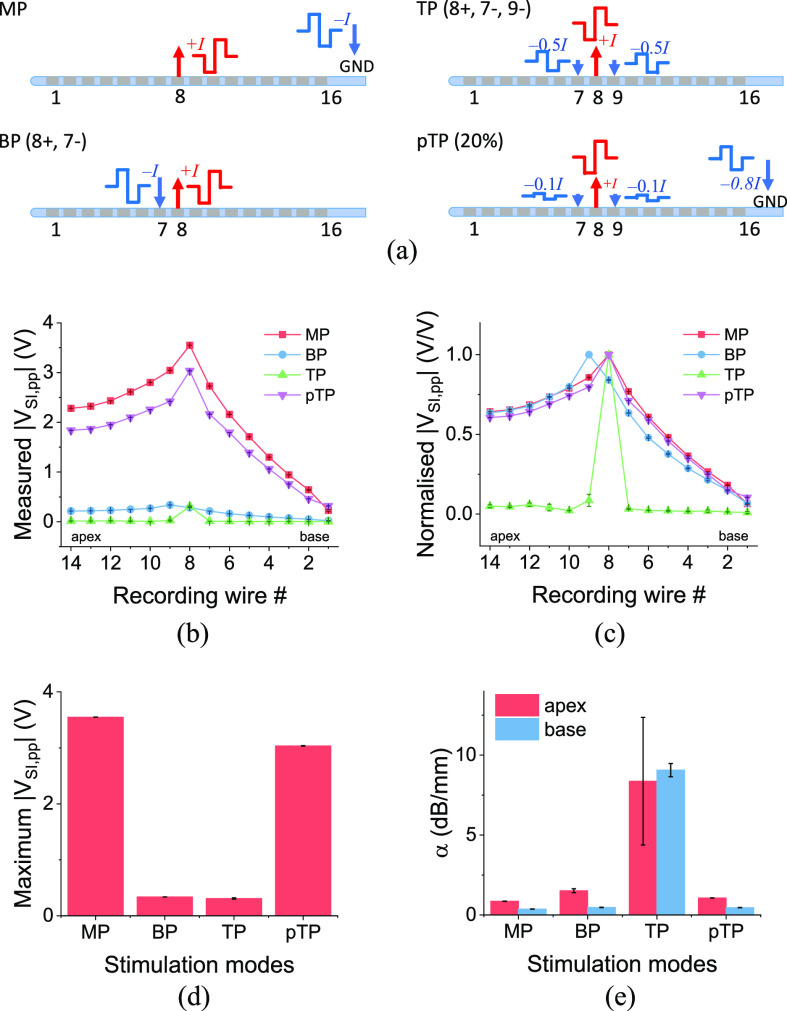
(a) Schematics of current stimulus injection and sinking under different stimulation modes, namely MP, BP, TP, and pTP. (b, c) The peak-to-peak SIV distribution in the artificial cochlea, (b) as measured and (c) normalised, under different stimulation modes. (d) The maximum absolute measured peak-to-peak SIV and (e) the stimulation focusing under different stimulation modes - an exponential decay fitting was used to extract the decay parameter (*α*) expressed in units of decibel per millimetre (dB/mm).

The stimuli were programmed with the Bionic Ear Data Collection System (BEDCS) research software from Advanced Bionics. All the stimulus pulses were charge-balanced, which is ensured by BEDCS, to prevent residual charge that can cause tissue damage [28]. We tested MP, BP, TP and pTP stimulation modes with biphasic pulses, all using 800 μA amplitude stimulation with each phase lasting 32 μs, centered on CI stimulating electrode 8, and aligned visually with the 8^th^ recording wire in the artificial cochlea. These stimulation modes are schematically depicted in Fig. 3(a). For the central (8^th^) electrode of the CI, the pulse was cathodic-leading (Fig. 2(a)); for the current sinking electrodes, the pulses were anodic-leading (Fig. 3(a)), and the amplitudes were determined according to the stimulation modes. In this study, only the SIV distributions for CI stimulating electrode 8 was presented, which is a representative case for other electrodes and other cochlea models with different geometries and resistances. To provide clinical information specific to a patient, a cochlea model with same geometry and resistivity to that of an individual patient would have to be fabricated and the SIV distributions for each electrode need to be characterised to find how stimulation patterns affect electric fields in that particular cochlea, but we believe we can suggest general findings of interest to all patient geometries.

The EFI was also measured in the artificial cochlea model, using Volta software from Advanced Bionics. The measurement setup was the same as for clinical measurements, i.e., with an amplitude of 32 μA and phase duration of 36 μs. The EFI data in the artificial cochlea model were compared with those obtained from patients. The conduct of this study was approved by the Human Biology Research Ethics Committee, University of Cambridge (Project No. HBREC.2019.42) on 8 January 2020, and by the Research & Development Department, Cambridge University Hospitals NHS Foundation Trust (Project No. A095451) on 11 May 2020.

### C. SIV Measurement Setup

The artificial cochlea lumen was filled with 1% w/v sodium chloride (NaCl) solution, and immersed in a saline bath with the same NaCl concentration. The CI was inserted into the lumen to match the clinically implanted patient scenario (Fig. 2(b)). The saline filling of the tube was designed to mimic closely the electrical conductivity of the perilymph [29]. The ground electrode from the CI was also immersed in the saline bath. The resistors were grounded to the CI ground electrodes with electrical wires.

The voltage measurements across the resistors were recorded with a Teledyne LeCroy HDO4054A-MS oscilloscope. The sampling rate was 1 GHz. The results were transmitted to a LabVIEW program and conditioned with a digital Butterworth low-pass filter at 6.25 MHz to remove the radio frequency noises from the CI processor. The peak-to-peak voltage between the two phases was extracted as a quantitative measurement for the degree of SIV. The measurements were conducted three times and the standard deviations were calculated. We present the average SIV measurements with standard deviations as error bars. Normalized values were calculated with respect to the highest value recorded. Normalization was used because MP stimulations demonstrated good linearity of SIV growth with stimulus amplitude levels, and similarly with other stimulus modes (Fig. S3).

### D. Spread-Induced Voltage (SIV) Signal

To quantify the stimulus spread, we measured the SIV signals (*V*_SI_) using the CI to generate current stimuli and the implanted silver wires in the model wall to record the voltage signals, illustrated schematically in Fig. 1(c) and as photographed in Fig. 1(d). We used the BEDCS software from Advanced Bionics to generate a biphasic charge-balanced square wave pulse, delivered by the chosen CI electrode. An example of stimulating and recording is shown in Fig. 2(a), where stimulating electrode number 8 and recording wire 8 were used. The measured SIV signal demonstrated a resistor-capacitor circuit like time course, i.e., not a truly square shape. This can be explained by the complex impedance of the saline and the ground electrode, which normally contain both resistance and capacitance components [30], [31]. To measure the degree of spread along the cochlea, we extracted the peak-to-peak voltage (*V*_SI,pp_) from the measured SIV waveforms at all the recording wires (Fig. 2(b)). All the *V*_SI,pp_ data in the main text are shown on a linear scale as with an *in-vitro* study, and the same data presented on a logarithmic scale more related to hearing perception can be found in Fig. S10.

We acknowledge that using the SIV signal to estimate the current spread distribution and to describe stimulation focusing is a preliminary approach, since it does not inform us about to neural responses to the stimulation. Neural activation is generally thought to be best predicted by the activation function [24], which is the second derivative of the voltage distribution along the nerve. We are not actually measuring that, but models such as by Kalkman et al [32] imply that having a larger voltage in the cochlea results in greater neuronal activation. In order to understand neural activities in response to stimulation, computational neural models could be used with the SIV data measured in the physical model to try to model voltage patterns along the peripheral processes or central axon. Alternatively, biological neurons can be cultured on the sensing electrodes of the cochlea model, so that neural activities to this SIV signal could be obtained directly. This tool is not completely satisfactory as cultured neurones may not have the same response patterns as “in-situ” spiral ganglion cells. In this study, we focus on the electrical characteristics of the stimulation rather than the resulting neural responses.

## III. Results and Discussion

### A. 3D-Printed Cochlea Model

The 3D-printed unwrapped artificial cochlea model demonstrated electrical spread characteristics similar to real cochleae. As mentioned above, to simplify the cochlea structure, we 3D-printed an unwrapped artificial cochlea model (Fig. 1(a)), with a similar geometry to the cochlear lumen in a real cochlea [26] (Fig. 1(b)). Based on computational modeling, the unwrapped and spiral models appear to have similar electrical spread characteristics (Fig. S13). To simulate the resistance that would normally allow some current to flow out of the cochlear lumen through the bony walls of real cochleae [23], we connected resistors along the length of our artificial unwrapped cochlea connecting the lumen to the surrounding saline bath, in which the casing ground electrode was immersed (“transverse” resistors) since the resin we used for the 3D printed artificial cochlea is not as electrically conductive as real cochlear bone (Fig. 1(c)). We compared a range of resistance values and “tuned” them to calibrate the electrical characteristics of the artificial cochlea so that we achieved SIV profiles similar to those measured in a typical *in-vivo* patient profile measured using a CI (in this case measured using the EFI function from Advanced Bionics, as most of our *in-vitro* experiments were also performed with an Advanced Bionics CI). That is, we aimed to produce the same SIV profile as in real cochlea when measured using the intra-cochlear electrodes in both cases, and then evaluated the voltage spread using our own recording electrodes, which included those placed close to the stimulating electrode. The same resistance values were used for all transverse resistors since in real life, the distances from the recording locations to the remote casing ground electrode are relatively similar, at least in MP mode, with likely the same tissue pathways, and therefore we would expect impedances from the cochlear lumen to the ground to be roughly similar. Therefore, the transverse resistances to ground for MP stimulation are likely to be in the same resistance range but with some variations. We simplified the resistive network and used an identical resistance for all the resistors.

We used the MP mode and biphasic pulses (Fig. 2(a)) to match our SIV measurements and extracted the peak-to-peak voltage (*V*_SI,pp_), since this is the configuration used clinically for EFI measurements. Note that a time-dependent increase was observed in the *V*_SI_ waveform (Fig. 2(a)), which could be possibly attributed to the capacitive impedance elements in the saline [33] and also saline/wire interface impedances. Despite the real possibility of saline/wire interface impedances confounding measurements, they are likely to be insignificant in impact on the SIV waveforms recorded across the transverse resistors. There are three reasons for this assertion, which can be found in the Supplementary Materials.

The *in-vivo* EFI demonstrated an inverted-V-shaped profile, as depicted in Fig. 2(b)(see the *x*-axis mapping of *in-vivo* EFI data in Supplementary Materials). Without transverse resistors, the normalized (with respect to the highest value recorded) SIV profile was quite flat at the apical side. This was because, given the insulating resin cochlea wall, there was a more restricted current pathway at the apex of the cochlea than at the base, and therefore the *V*_SI,pp_ at the apical side was at nearly the same level as at the stimulating electrode. In general, the apical part of the cochlea has a much smaller lumen than the basal side and so there is less conductive electrolyte here for longitudinal charge spread, and EFI or TIM measurements in living subjects generally also show a much flatter SIV at the apical than at the basal end (see 5 *in-vivo* EFI examples in Fig. S7). When transverse resistors were added, the measured SIV starts show an inverted-V-shaped profile, with a more significant decrease in *V*_SI,pp_ at the basal than at the apical side. Empirically, SIV measurements with 47kΩ transverse resistors demonstrated reasonable agreement with the *in-vivo* EFI. This resistance value is also in the same magnitude range of the transverse resistors from *in-vivo* measurements [21]. Note that using identical resistances for all resistors is not an ideal solution, but it tunes the shape of *V*_SI,pp_ to match *in-vivo* EFI to a first approximation quite well. Given the fact that *in-vivo* EFI is a result of the combination of individual cochlear geometry and resistivity, it would be impossible to fabricate an *in-vitro* cochlea model that has completely identical EFI to any one individual using just the average human cochlea size and geometry. For future development of *in-vitro* cochlea models, it is essential to obtain linked cochlear geometry information and EFIs from patients. As a simplification, we used 47kΩ resistors in the 3D printed cochlea model to electrically mimic a real cochlea.

To validate the 3D-printed cochlea model, we simulated the model with COMSOL by importing the CAD file used for 3D printing of it, so that theoretically, we have the same geometry design for both 3D-printed *in-vitro* model and computational COMSOL model. As seen in Fig. 2(c), the *in-vitro* model and COMSOL model demonstrate similar *V*_SI,pp_ distribution with different resistor levels. These results also indicate that COMSOL simulation can be used to find the transverse resistances variations to better fit an *in-vivo* EFI, instead of trial and error with different resistances. However, since EFI results from a combination result of cochlea geometry and resistivity, and the cochlea geometry information that linked to the patient was missing, it is not very meaningful to find the exact transverse resistances here without the exact geometry. As an additional validation step, we measured the EFI in the artificial model as well. Despite some higher apical EFI data, the artificial model shows a similar EFI profile (the average of three measurements to reduce noises) to *in-vivo* intra-op data (Fig. 2(d) and S7). The apical EFI profile discrepancy indicates the importance of variations in transverse resistances for future development of artificial models. With COMSOL simulation, we found that the selections of transverse resistances can be optimized to better fit in-vivo EFI data (Fig. S8 and Table S2), with root-mean-square errors below 7%. The combinations of transverse resistances in COMSOL simulation were selected manually and empirically, and can be further optimized and automated using some genetic algorithms or machine learning algorithms. In turn, this will help the design of the transverse resistor network in the in-vitro models.

### B. Comparison Among Classic Stimulation Modes

We found different stimulation modes demonstrated a trade-off between maximum *V*_SI,pp_ and stimulation focusing. Again, we are assuming that *V*_SI,pp_ plays some role in deciding whether a neuron crosses the activation threshold for firing, even though it may not determine the site of activation, which is likely determined by the activation function [24], then the maximum *V*_SI,pp_ (Fig. 3(d)) may well be associated with neuronal firing rate [32]. Firing rate and spread would be related to the loudness that CI recipients would perceive (if there were nerve cells in that region), whereas the stimulation focusing is related to the extent of spread of activation among different parts of auditory nerves for a given stimulation electrode, i.e., the bandwidth of the sound. As shown in Fig. 3(b)–(d), MP stimulation mode provided the highest maximum *V*_SI,pp_ at the 8^th^ recording wire, followed by pTP, BP and TP stimulations. The higher maximum *V*_SI,pp_ means that a lower current stimulus amplitude is likely to be needed to achieve the same hearing threshold, and therefore this mode provides the lowest power consumption for a CI. MP stimulation mode does not contain an intracochlear current sinking electrode, so it maximizes the voltage built up between the cochlea and the ground electrode. BP stimulation, by definition, uses one intracochlear current sinking electrode, so that the voltage at the recording site is reduced around this electrode asymmetrically depending on whether the sink electrode is located apical or basal to the stimulating electrode. The sink electrode was pulsed with a biphasic pulse of the opposite polarity to the stimulating electrode. TP stimulation mode used two current sinking electrodes, and therefore the voltage was reduced from both the apical and basal sides of cochlea lumen. Since pTP stimulation can be regarded as a combination of MP and TP stimulations, it demonstrated a maximum *V*_SI,pp_ between the values obtained from MP and TP stimulation modes.

Note that we also recorded some SIV waveforms in multipolar (BP, TP) stimulation modes with an opposite polarity to the main stimulating electrode, i.e., having an anodic-leading rather than cathodic-leading profile (Fig. S5). This was because we used current sinking electrodes with an opposite polarity in multipolar stimulations, and the voltage induced on the recording electrodes can sometimes be dominated by these current sinks rather than the current source. This has implications for auditory nerve stimulation, especially if there is a larger residual neural population closer to the current sink than the current source, because the auditory nerve is not equally sensitive to anodic and cathodic current [16], [34]–[37]. The details of this are discussed in the Supplementary Materials.

In terms of the stimulation focusing, the four stimulation modes showed a reverse trend to their SIV amplitude trend, as shown in Fig. 3(e). MP stimulation produced the broadest SIV profile, whereas TP stimulation gave the most focused stimulation. BP stimulation seemed to have a similar focusing effect as MP stimulation, although it was quite asymmetrical (Fig. S6). More discussion about BP stimulations can be found in Supplementary Materials.

### C. Intra vs. Extra-Cochlear Current Sinks in pTP Modes

A larger percentage of current sinking to intracochlear electrodes in pTP mode improved stimulation focusing at the cost of a lower maximum *V*_SI,pp_. Since pTP stimulation is an intermediary between MP and TP stimulation, with a trade-off between maximum *V*_SI,pp_ and stimulation focusing, we further investigated pTP stimulation to find an optimized pTP trade-off between SIV voltage and focusing. We used *σ* as the percentage of stimulation current sinking into the intracochlear electrodes and varied the parameter from 0 to 100%.

**Fig. 4.**
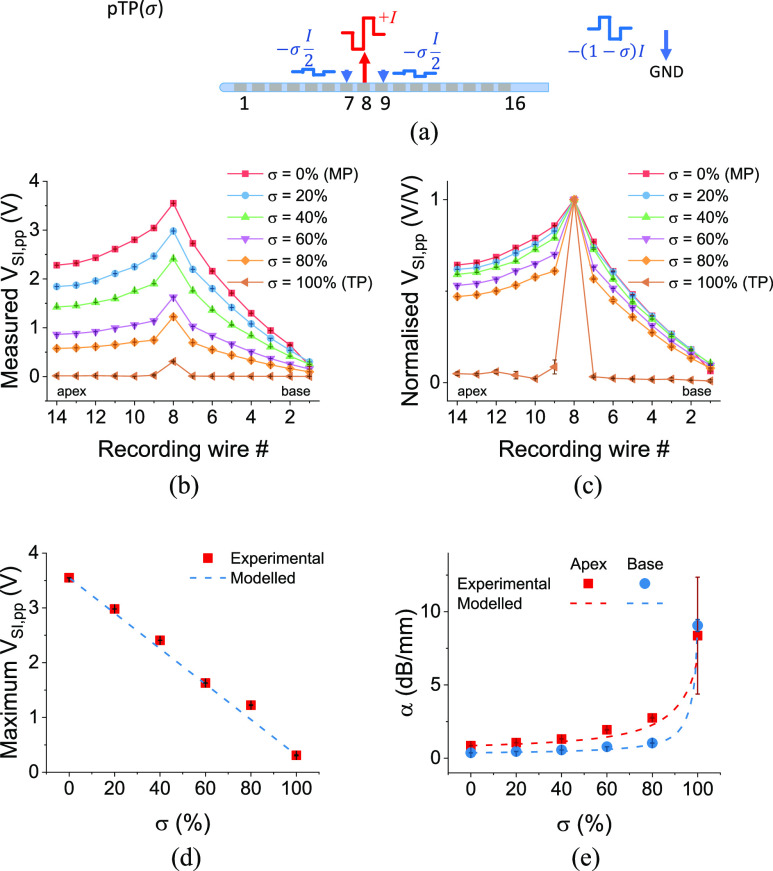
(a) A schematic of current stimulus injection and sinking under pTP (*σ*) stimulation. When *σ* = 0, pTP stimulation mode is MP stimulation by definition, whereas when *σ* = 100%, it is equivalent to TP stimulation. (b, c) The peak-to-peak SIV distribution in the artificial cochlea, (b) as measured and (c) normalised, under different pTP modes, where *σ* denotes the percentage of TP mode. (d) The maximum measured peak-to-peak SIV and (e) the stimulation focusing as a function of *σ*.

Assessing the effect of current sinking to intracochlear electrodes by means of varying *σ* in pTP stimulation (Fig. 4(a)), the data largely showed similar trends to those previously discussed (Fig. 4(b),(c)). A larger percentage of current sinking to intracochlear electrodes improved the stimulation focusing at the cost of sacrificing the maximum *V*_SI,pp_. As seen in Fig. 4(c), the maximum peak-to-peak SIVs at the 8^th^ recording wire appeared to vary with *σ* approximately linearly over the range *σ* = 0% to 100%. According to Wu et al [25], the potential field for pTP mode can be regarded as a linear sum of those from the main and flanking electrodes. This can be described as:

\begin{equation*}
{V_{SI,pp,pTP}}\ \left({\sigma,i} \right) = \left({1 - \sigma } \right)\ {V_{SI,pp,MP}}\left(i \right) + \sigma {V_{SI,pp,TP}}\left(i \right)\tag{1}
\end{equation*}

where *V*_SI,pp,pTP_, *V*_SI,pp,MP_ and *V*_SI,pp,TP_ stand for peak-to-peak SIV amplitude under pTP, MP and TP modes, respectively, at the *i*^th^ recording wire. From equation 1, which is linear for *σ*, we modelled the maximum *V*_SI,pp_ at different *σ*. As shown in Fig. 4(d), the experimental and modelled data were in a good agreement, again confirming that there is good linearity of the measured maximum *V*_SI,pp._ However, for the same range, stimulation focusing appeared to be non-linear (Fig. 4(e)). When *σ* = 100%, a dramatic increase in the stimulation focusing and reduction in the *V*_SI,pp_ was measured. For *σ* = 0% to 80%, there was always some current being drawn towards the extra-cochlear ground, whereas for *σ* = 100%, the ground was fully intra-cochlear. The confinement of the current pathway to solely intra-cochlear electrodes appeared therefore to have a significant effect on focusing. To understand the dramatic change from *σ* = 80% to 100%, we modelled the stimulation focusing and extracted the parameter *α* using equations 1 and S1. As shown in Fig. 4(e), the most dramatic change in stimulation focusing happened when *σ* exceeded 95%.

### D. Different Current Source/Sink Distances in TP+n Modes

**Fig. 5.**
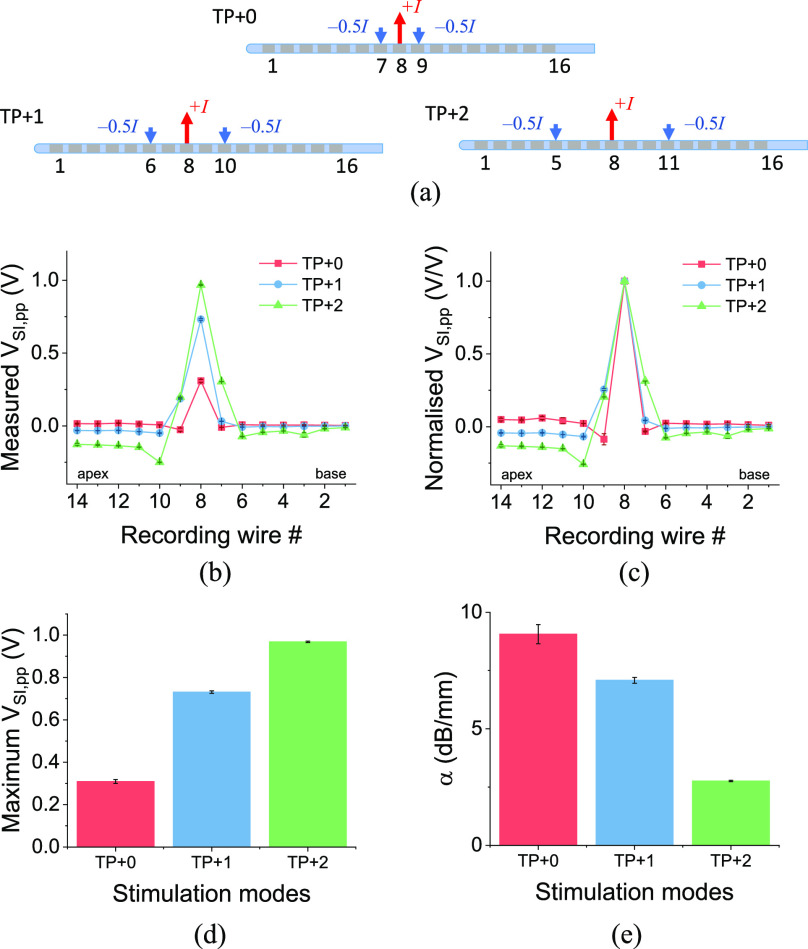
(a) Schematics of current stimulus injection and sinking under different configurations of TP+*n* stimulation. (b, c) The peak-to-peak SIV distribution in the artificial cochlea, (b) as measured and (c) normalised, under different TP+*n* modes. (d) The maximum measured peak-to-peak SIV and (e) the stimulation focusing under different TP+*n* modes.

Increased intracochlear current sinking electrode distance in TP+*n* stimulation modes decreases power consumption without sacrificing stimulation focusing, where the term +*n* refers to the number of electrodes between the stimulating electrode and current sinking electrode. With regards to the effect of changing distance between stimulating and current sinking electrodes for TP modes (Fig. 5(a)), our results show that various configurations demonstrated enhanced stimulation focusing, but at the cost of lowered *V*_SI,pp_ with reducing the distance *n* (Fig. 5(b),(c)). Actually, TP+2 showed a profile that was almost as focused as in TP+0 mode, but with a much higher *V*_SI,pp_ level (Fig. 5(d),(e)). Comparing with MP mode, the maximum *V*_SI,pp_ in TP+2 mode was 3.5 times lower, and hence increased power would be needed to match the two modes for stimulating auditory nerves to firing threshold. Despite these power costs, the TP+2 mode has significantly better stimulation focusing when compared to MP mode.

These results support the use of TP stimulation modes with current sinking electrodes further separated than just adjacent to the stimulating electrodes to provide reduced power consumption without sacrificing focusing inordinately. Recommendations between TP+*n* and MP modes will depend on whether the priority is power efficiency or limiting stimulation spread. Whether current spread is still narrower in TP+*n* mode at higher stimulation levels to achieve a similar *V*_SI,pp_ to MP mode needs to be investigated further, possibly by incorporating a neural model and/or CI patient study.

### E. Combining Advantages of pTP and TP+n Modes

Modelling for pTP+*n* mode shows stimulation amplitude and focusing can be optimized by combining different stimulation modes and changing intracochlear current sinking configurations. Based on the results from TP+*n* and MP modes, we simulated the stimulation levels and focusing abilities in pTP+*n* modes. Here, our hypothesis was that the results in pTP+*n* modes would be a linear combination of TP+*n* and MP modes, for which we found good agreement with the experimental data in pTP stimulation.

**Fig. 6.**
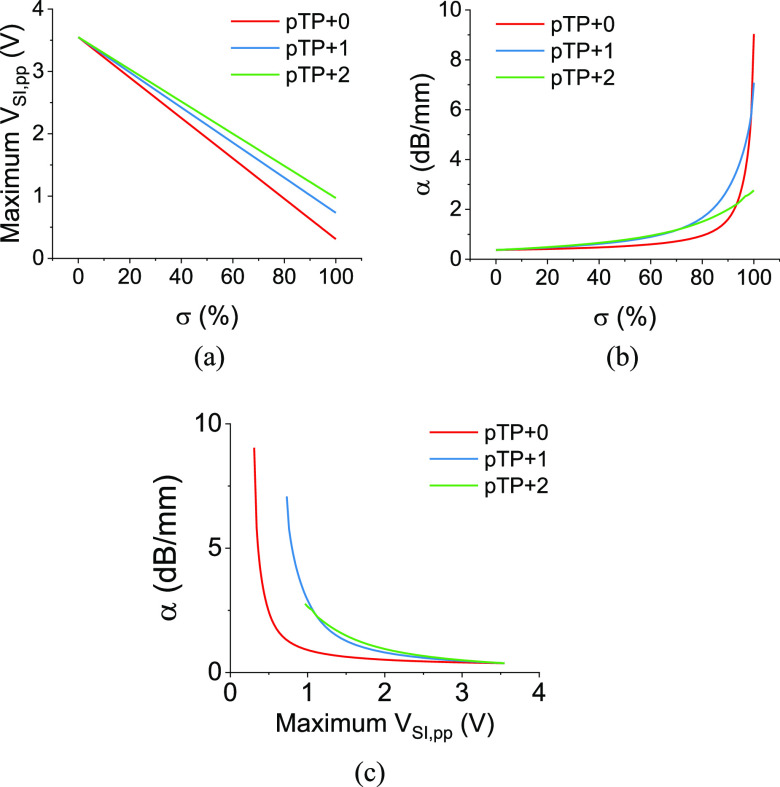
(a) Modelling of the maximum peak-to-peak SIV and (b) the stimulation focusing under different pTP+*n* modes. (c) The modelled relationship between stimulation focusing and the maximum peak-to-peak SIV under different pTP+*n* modes.

In terms of stimulation levels, the maximum *V*_SI,pp_ showed good linearity with respect to the percentage of TP+*n* contribution (Fig. 6(a)), which was expected. Contrary to this, the stimulation focusing was modelled and found to vary nonlinearly with *σ* (Fig. 6(b)). For all the fittings, the coefficient of determination was larger than 0.96 (Fig. S9). Note that there were some intercepts in the curves with different distances of current sinking electrodes from the centering electrode. When *σ* is below ∼70%, pTP+1 and pTP+2 modes could deliver more focused stimulation than pTP+0 modes at the same *σ*, with slightly higher *α* for pTP+2. With *σ* being between 70% and 99%, pTP+1 demonstrated the most focused stimulation compared to the other pTP modes. There is only a small window, when *σ* is greater than 99%, for which the closest current sink to the stimulus electrode has the best focusing ability. Nevertheless, *σ* is a parameter of the stimulation configuration, and it would be meaningful to investigate the relationship between power consumption and stimulation focusing.

As seen in Fig. 6(c), the pTP+0 modes deliver much lower *V*_SI,pp_ at the same stimulus level, with the benefit of a slightly better focusing effect. The pTP+1 and pTP+2 modes seem to be quite similar in this regard, except that pTP+1 allows higher stimulation focusing that is comparable to that in the TP+0 mode. Though these results cannot directly prove that pTP+1 provides the best compromise between stimulation threshold and focusing for all CI recipients, they indicate some trends and compromises that should be considered when choosing various stimulation configurations. In addition, it is suggested that, beyond a certain level, increased focusing cannot be differentiated by the auditory nerve [9], so it may be worth combining auditory nerve stimulation models with these experimental results to assess whether the TP+*n* mode can deliver improved focusing compared to other modes. It is also possible that too much current focusing might not recruit enough neurons for a reasonable comfortable loudness perception level, and current injection may need to be increased to loudness balance different stimulation types in real life before comparisons of distinguishing ability for speech or spectral patterns can be made.

It may also be interesting to culture cochlear spiral ganglion neurons in the in-vitro model, especially on the electrodes of recording wires to directly record neural excitation rates under spread stimulations.

## IV. Conclusion and Future Work

We have presented a novel platform, i.e., 3D-printed unwrapped “artificial cochlea” with instrumented recording wires, to measure intracochlear current spread and compare different stimulation modes commonly investigated for cochlear implants. The results (summarized in Table S1) provided quantitative evidence of the differences in SIV distribution among MP, BP, TP stimulation modes with different configurations. Generally, there are trade-offs between the stimulation levels and focusing when comparing different stimulation modes. In addition, apical and basal current sinks can greatly affect SIV distributions in the cochlea. Moreover, we found that there is an optimum in the distance between stimulating and sinking electrodes. This platform allows customizing a 3D-printed cochlea model with cochlea geometry and electrical properties tunable to match a real cochlea, if geometry (from CT/MRI scans) and EFI (from a CI) can be available with sufficient detail, thus enabling *in-vitro* study on electrical stimulus spread for a CI user.

In future studies, we aim to investigate other advanced stimulation modes such as phased array and current steering. In addition, we envision advanced bioprinting technology will allow us to build artificial cochleae that simulate the 3D shape of human cochleae, and can be instrumented with a dense network of recording wires by microfabrication to visualize current spread distributions at a high resolution. We also wish to develop a neural model and/or to culture cochlear spiral ganglion neurons in the in-vitro model that could link the current spread distribution to how neurons respond to electrical stimuli and what CI users might hear.

## Supplementary Material

10.1109/TBME.2021.3059302 An Instrumented Cochlea Model for the Evaluation of Cochlear Implant Electrical Stimulus Spread Cochlear implants use electrical stimulation of the auditory nerve to restore the sensation of hearing to deaf people. Unfortunately, the stimulation current spreads extensively within the cochlea, resulting in “blurring” of the signal, and hearing that is far from normal. Current spread can be indirectly measured using the implant electrodes for both stimulating and sensing, but this provides incomplete information near the stimulating electrode due to electrode-electrolyte interface effects. Here, we present a 3D-printed “unwrapped” physical cochlea model with integrated sensing wires. We integrate resistors into the walls of the model to simulate current spread through the cochlear bony wall, and “tune” these resistances by calibration with an in-vivo electrical measurement from a cochlear implant patient. We then use this model to compare electrical current spread under different stimulation modes including monopolar, bipolar and tripolar configurations. Importantly, a trade-off is observed between stimulation amplitude and current focusing among different stimulation modes. By combining different stimulation modes and changing intracochlear current sinking configurations in the model, we explore this trade-off between stimulation amplitude and focusing further. These results will inform clinical strategies for use in delivering speech signals to cochlear implant patients. https://doi.org/10.1109/TBME.2021.3059302

## References

[1] Q. J. Fu, “Temporal processing and speech recognition in cochlear implant users,” Neurorep., vol. 13, no. 13, pp. 1635–1639, Sep. 2002. 10.1097/00001756-200209160-00013 12352617

[2] O. Macherey, and R. P. Carlyon, “Cochlear implants,” Curr. Biol., vol. 24, no. 18, pp. R878–R884, 2014. 25247367 10.1016/j.cub.2014.06.053

[3] J. A. Grange , “Cochlear implant simulator with independent representation of the full spiral ganglion,” J. Acoust. Soc. Amer., vol. 142, no. 5, pp. EL484–EL489, Nov. 2017. 29195445 10.1121/1.5009602

[4] M. D. Fletcher, S. R. Mills, and T. Goehring, “Vibro-tactile enhancement of speech intelligibility in multi-talker noise for simulated cochlear implant listening,” Trends Hear.*,* vol. 22, 2018, Art. no. 2331216518797838. 10.1177/2331216518797838 PMC6144588 30222089

[5] J. B. Firszt , “Recognition of speech presented at soft to loud levels by adult cochlear implant recipients of three cochlear implant systems,” Ear Hear.*,* vol. 25, no. 4, pp. 375–387, Aug. 2004. 15292777 10.1097/01.aud.0000134552.22205.ee

[6] F. G. Zeng , “Development and evaluation of the nurotron 26-electrode cochlear implant system,” Hear. Res., vol. 322, pp. 188–199, 2015. 25281795 10.1016/j.heares.2014.09.013

[7] L. M. Friesen , “Speech recognition in noise as a function of the number of spectral channels: Comparison of acoustic hearing and cochlear implants,” J. Acoust. Soc. Amer., vol. 110, no. 2, pp. 1150–1163, Aug. 2001. 11519582 10.1121/1.1381538

[8] Q. J. Fu, and G. Nogaki, “Noise susceptibility of cochlear implant users: The role of spectral resolution and smearing,” JARO-J. Assoc. Res. Otolaryngol., vol. 6, no. 1, pp. 19–27, 2005. 10.1007/s10162-004-5024-3 PMC2504636 15735937

[9] A. G. Srinivasan, D. M. Landsberger, and R. V. Shannon, “Current focusing sharpens local peaks of excitation in cochlear implant stimulation,” Hear. Res., vol. 270, no. 1–2, pp. 89–100, 2010. 20850513 10.1016/j.heares.2010.09.004 PMC2997903

[10] T. Goehring, J. G. Arenberg, and R. P. Carlyon, “Using spectral blurring to assess effects of channel interaction on speech-in-noise perception with cochlear implants,” JARO - J. Assoc. Res. Otolaryngol., 2020. 10.1007/s10162-020-00758-z PMC7445227 32519088

[11] Q. Tang, R. Benítez, and F.-G. Zeng, “Spatial channel interactions in cochlear implants,” J. Neural Eng., vol. 8, no. 4, pp. 1–14, 2011. 10.1088/1741-2560/8/4/046029 PMC3190971 21750370

[12] Z. Zhu , “Cochlear-implant spatial selectivity with monopolar, bipolar and tripolar stimulation,” Hear. Res., vol. 283, no. 1–2, pp. 45–58, Jan. 2012. 22138630 10.1016/j.heares.2011.11.005 PMC3277661

[13] M. L. Hughes, Objective Measures in Cochlear Implants. San Diego, CA, USA: Plural Publishing, 2012.

[14] A. A. Saoji , “Masking patterns for monopolar and phantom electrode stimulation in cochlear implants,” Hear. Res., no. 298, pp. 109–116, 2013. 10.1016/j.heares.2012.12.006 PMC3755121 23299125

[15] R. P. Carlyon , “Evaluation of a cochlear-implant processing strategy incorporating phantom stimulation and asymmetric pulses,” Int. J. Audiol., vol. 53, no. 12, pp. 871–879, 2014. 25358027 10.3109/14992027.2014.932024 PMC4266076

[16] O. Macherey, J. M. Deeks, and R. P. Carlyon, “Extending the limits of place and temporal pitch perception in cochlear implant users,” J. Assoc. Res. Otolaryngol., vol. 251, pp. 233–251, 2011. 10.1007/s10162-010-0248-x PMC3046333 21116672

[17] V. Sankarasubramanian , “Triple leads programmed to perform as longitudinal guarded cathodes in spinal cord stimulation: A modeling study,” Neuromodulation, vol. 14, no. 5, pp. 401–411, 2011. 21854494 10.1111/j.1525-1403.2011.00383.x

[18] L. H. M. Mens, “Advances in cochlear implant telemetry: Evoked neural responses, electrical field imaging, and technical integrity,” Trends Amplif., vol. 11, no. 3, pp. 143–159, 2007. 17709572 10.1177/1084713807304362 PMC4111364

[19] A. Kral , “Spatial resolution of cochlear implants: The electrical field and excitation of auditory afferents,” Hear. Res., vol. 121, no. 1–2, pp. 11–28, Jul. 1998. 9682804 10.1016/s0378-5955(98)00061-6

[20] F. J. Vanpoucke, A. J. Zarowski, and S. A. Peeters, “Identification of the impedance model of an implanted cochlear prosthesis from intracochlear potential measurements,” IEEE Trans. Biomed. Eng., vol. 51, no. 12, pp. 2174–2183, Dec. 2004. 15605865 10.1109/TBME.2004.836518

[21] F. Vanpoucke , “The facial nerve canal: An important cochlear conduction path revealed by clarion electrical field imaging,” Otol. Neurotol., vol. 25, no. 3, pp. 282–289, 5 2004. 15129106 10.1097/00129492-200405000-00014

[22] Q. Mesnildrey , “Impedance measures for a better understanding of the electrical stimulation of the inner ear,” J. Neural Eng., vol. 16, no. 1, Feb. 2019. 10.1088/1741-2552/aaecff 30523898

[23] J. J. Briaire, and J. H. M. Frijns, “Field patterns in a 3D tapered spiral model of the electrically stimulated cochlea,” Hear. Res., vol. 148, no. 1–2, pp. 18–30, Oct. 2000. 10978822 10.1016/s0378-5955(00)00104-0

[24] F. Rattay, R. N. Leao, and H. Felix, “A model of the electrically excited human cochlear neuron. II. Influence of the three-dimensional cochlear structure on neural excitability,” Hear. Res., vol. 153, no. 1–2, pp. 64–79, 2001. 11223297 10.1016/s0378-5955(00)00257-4

[25] C. C. Wu, and X. Luo, “Current steering with partial tripolar stimulation mode in cochlear implants,” JARO - J. Assoc. Res. Otolaryngol., vol. 14, no. 2, pp. 213–231, Apr. 2013. 23250685 10.1007/s10162-012-0366-8 PMC3660915

[26] M. Thorne , “Cochlear fluid space dimensions for six species derived from reconstructions of three-dimensional magnetic resonance images,” Laryngoscope, vol. 109, no. 10, pp. 1661–1668, 1999. 10522939 10.1097/00005537-199910000-00021

[27] A. B. Corporation.“Surgeon's manual for hifocus helix and hifocus 1j electrodes,” 2004.

[28] K. L. Rodenhiser, and F. A. Spelman, “A method for determining the driving currents for focused stimulation in the cochlea,” IEEE Trans. Biomed. Eng., vol. 42, no. 4, pp. 337–342, Apr. 1995. 7729833 10.1109/10.376127

[29] J. S. Binette , “Tetrapolar measurement of electrical conductivity and thickness of articular cartilage,” J. Biomech. Eng., vol. 126, no. 4, pp. 475–484, Aug. 2004. 15543865 10.1115/1.1785805

[30] S. Dovancescu , “Monitoring thoracic fluid content using bioelectrical impedance spectroscopy and cole modeling,” J. Electr. Bioimpedance, vol. 8, no. 1, pp. 107–115, 2017.

[31] L. F. Lima , “Electric impedance of aqueous KCl and nacl solutions: Salt concentration dependence on components of the equivalent electric circuit,” J. Mol. Liq., vol. 241, pp. 530–539, 2017.

[32] R. K. Kalkman, J. J. Briaire, and J. H. M. Frijns, “Current focussing in cochlear implants: An analysis of neural recruitment in a computational model,” Hear. Res., vol. 322, pp. 89–98, 2015. 25528491 10.1016/j.heares.2014.12.004

[33] Q. Huang , “The effect of electrolyte concentration on electrochemical impedance for evaluating polysulfone membranes,” Environ. Sci. Water Res. Technol., vol. 4, no. 8, pp. 1145–1151, 2018.

[34] R. P. Carlyon, J. M. Deeks, and O. Macherey, “Polarity effects on place pitch and loudness for three cochlear-implant designs and at different cochlear sites,” J. Acoust. Soc. Amer., vol. 134, no. 1, pp. 503–509, Jul. 2013. 23862825 10.1121/1.4807900

[35] O. Macherey , “Higher sensitivity of human auditory nerve fibers to positive electrical currents,” JARO - J. Assoc. Res. Otolaryngol., vol. 9, no. 2, pp. 241–251, Jun. 2008. 18288537 10.1007/s10162-008-0112-4 PMC2413083

[36] F. Rattay, “The basic mechanism for the electrical stimulation of the nervous system,” Neuroscience, vol. 89, no. 2, pp. 335–346, Mar. 1999. 10077317 10.1016/s0306-4522(98)00330-3

[37] O. Macherey, and R. P. Carlyon, “Place-pitch manipulations with cochlear implants,” J. Acoust. Soc. Amer., vol. 131, no. 3, pp. 2225–2236, 2012. 22423718 10.1121/1.3677260 PMC3383798

